# Sintering Behavior, Thermal Expansion, and Environmental Impacts Accompanying Materials of the Al_2_O_3_/ZrO_2_ System Fabricated via Slip Casting

**DOI:** 10.3390/ma14123365

**Published:** 2021-06-17

**Authors:** Justyna Zygmuntowicz, Magdalena Gizowska, Justyna Tomaszewska, Paulina Piotrkiewicz, Radosław Żurowski, Marcin Wachowski, Katarzyna Konopka

**Affiliations:** 1 Faculty of Materials Science and Engineering, Warsaw University of Technology, 141 Woloska Street, 02-507 Warsaw, Poland; paulina.piotrkiewicz.dokt@pw.edu.pl (P.P.); Katarzyna.Konopka@pw.edu.pl (K.K.); 2 Research Group of Special Ceramic Materials, Division of Ceramic and Concrete in Warsaw, Łukasiewicz Research Network—Institute of Ceramics and Building Materials, 9 Postępu Street, 02-676 Warsaw, Poland; m.gizowska@icimb.pl; 3 Instytut Techniki Budowlanej, Ksawerów 21, 02-656 Warsaw, Poland; j.tomaszewska@itb.pl; 4 Faculty of Chemistry, Warsaw University of Technology, 3 Noakowskiego Street, 00-664 Warsaw, Poland; rzurowski@ch.pw.edu.pl; 5 Faculty of Mechanical Engineering, Military University of Technology, 2 Gen. S. Kaliskiego Street, 00-908 Warsaw, Poland; marcin.wachowski@wat.edu.pl

**Keywords:** Al_2_O_3_/ZrO_2_, thermal expansion, sintering behavior, life cycle analysis, slip casting

## Abstract

This work focuses on research on obtaining and characterizing Al_2_O_3_/ZrO_2_ materials formed via slip casting method. The main emphasis in the research was placed on environmental aspects and those related to the practical use of ceramic materials. The goal was to analyze the environmental loads associated with the manufacturing of Al_2_O_3_/ZrO_2_ composites, as well as to determine the coefficient of thermal expansion of the obtained materials, classified as technical ceramics. This parameter is crucial in terms of their practical applications in high-temperature working conditions, e.g., as parts of industrial machines. The study reports on the four series of Al_2_O_3_/ZrO_2_ materials differing in the volume content of ZrO_2_. The sintering process was preceded by thermogravimetric measurements. The fabricated and sintered materials were characterized by dilatometric study, scanning electron microscopy, X-ray diffraction, and stereological analysis. Further, life cycle assessment was supplied. Based on dilatometric tests, it was observed that Al_2_O_3_/ZrO_2_ composites show a higher coefficient of thermal expansion than that resulting from the content of individual phases. The results of the life cycle analysis showed that the environmental loads (carbon footprint) resulting from the acquisition and processing of raw materials necessary for the production of sinters from Al_2_O_3_ and ZrO_2_ are comparable to those associated with the production of plastic products such as polypropylene or polyvinyl chloride.

## 1. Introduction

Ceramic materials, including ceramic–ceramic composites, have many desirable characteristics such as a resistance to aggressive environmental conditions [1,2,3,4], high hardness, and resistance to brittle fracture [5,6,7,8]. Thanks to these properties, ceramic materials are of great interest to the chemical, electromechanical, or fuel and energy industries. These materials can operate under conditions where plastic or metal components cannot always be used. Based on the literature, there are many methods of molding ceramic materials [9,10,11]. Among them are methods such as tape casting [12,13], gel casting [14,15], and slip casting [16,17] or stereolithography [18]. However, note that it is the choice of method for fabricating fittings that determines the properties of the resulting product.

In this study, the slip casting method was used to form ceramic fittings. It is a technology of fabricating samples that consists of pouring a ceramic mass, i.e., a suspension of ceramic particles in water, characterized by a high solid phase content and low viscosity. In this method, a mass of appropriately selected composition is poured into porous gypsum molds, in which moisture is removed from the slurry by capillary forces, resulting in a finished product [19]. The slip casting method makes it possible to obtain a product with complex shapes without using high pressures and temperatures. In addition, a ceramic slurry containing different grain sizes can be used in this technology. Furthermore, in slurries containing powder particles of a similar size there is no segregation of particles, which is a common problem when forming composites from loose masses. A significant advantage of slip casting is the low cost of the equipment used. Unfortunately, apart from the several advantages of this method, its disadvantage is that as the pores of gypsum molds become clogged with tiny particles of ceramic materials, the “suction force” of the mold decreases the period of durability of the mass and thus the quality of subsequent semi-finished products changes. Despite some inconveniences associated with slip casting, it is still quite commonly used to form various ceramic materials such as mulllite-ZrO_2_ [20,21], SiC [22], Si_3_N_4_ [23], ZrO_2_ [24,25], ZnO [26], or Al_2_O_3_ [27] composites, among others.

Note that new equipment and technologies are placing higher demand on technical ceramics. Therefore, the optimization of the composition of the individual components in ceramic samples based on experimental tests is still the subject of numerous research works. Several research works continue to be devoted to Al_2_O_3_, ZrO_2_, and Al_2_O_3_/ZrO_2_. Due to their favorable mechanical properties, these materials have become widespread in recent years and have acquired an almost infinite field of application. Selected examples include sliding components, machine parts, and tools. At the same time, due to the growing awareness of the effects of anthropogenic activities on the environment, increasing attention is being focused on environmental aspects [28,29]. As a consequence of the pro-environmental policies of the European Union, implemented as part of the economic transition towards the circular economy (CE) [30] and the European Green Deal [31], increasing restrictions are imposed on the industry in terms of permitted levels of emissions to air, water, and soil, as well as in terms of the amount of waste generated [30,31]. As a result, the drive to reduce the environmental footprint has become one of the critical challenges facing the industry [32,33].

This study focuses on the research dedicated to the fabrication and characterization of Al_2_O_3_/ZrO_2_ samples via slip casting. In the first stage of the study, thermogravimetric analysis was carried out to determine the temperatures at which complete thermal decomposition of the organic additives used during fabricating fittings occurs. Dilatometric tests were then carried out to determine coefficients of thermal expansion, which are of important practical significance. Note, here, that technical ceramics, including Al_2_O_3_, ZrO_2_, and Al_2_O_3_/ZrO_2_ composites, can all operate at high temperatures. A wide range of machine parts are made from this ceramic, including sliding elements, gaskets for heating nozzles, coil heaters, or elements for column heaters. Therefore, it is imperative to determine the changes in material volume due to temperature increases or decreases that play a crucial role in the operation of these devices. In the next stage of the study, the density of the sample was determined using the hydrostatic method. In order to determine the phase structure before and after the sintering process, an analysis was performed with an X-ray diffractometer. The microstructure of the samples was characterized based on microscopic observations made using a scanning electron microscope. Stereological methods were used to quantitatively characterize the microstructure of the fabricated samples, including a computer image analysis, to determine the effect of ZrO_2_ content on Al_2_O_3_ grain growth. The final stage of the research focused on determining the environmental impacts accompanying the process of forming ceramic elements. This research is original and has not yet been described in the literature for slip cast fabricated fittings. In addition, monitoring the environmental impact accompanying Al_2_O_3_ and ZrO_2_ fabrication of fittings creates the possibility of consciously developing new solutions, which are more environmentally friendly and require less energy. It is, therefore, a research area that is part of solving global problems facing humanity today.

The subject of this paper is Al_2_O_3_- and ZrO_2_-based ceramic materials. The research presented is interdisciplinary in nature, covering the field of the material’s engineering as well as its physic and environmental interactions. The study made it possible to assess the suitability of Al_2_O_3_-based materials with ZrO_2_ additives obtained via slip casting. The results obtained have made a significant contribution to the development of fundamental knowledge in the field of ceramic matrix composites.

## 2. Materials and Methods

Commercially available powders were used in the experiment: ZrO_2_ stabilized with 3% mol Y_2_O_3_ (TZ-PX-245; from Tosoh Co. (Tokyo, Japan) with a particle size of 0.04 µm and Al_2_O_3_ (TM-DAR; from Tamei Chemicals (Tokyo, Japan) with a particle size of 0.12 ± 0.3 µm. The densities of both powders were measured using a helium pycnometer (Accu Pyc II 1340 by Micromeritics, Norcross, GA, USA) and were 5.89 g/cm^3^ and 3.98 g/cm^3^, respectively. A complete characterization of the powders used in the experiment is presented in the study [34].

The slip casting method was used to mold the samples bodies. This method was chosen because of its ability to form elements of any geometry and the absence of toxic additives in the suspension used to fabricate the samples. Distilled water was used as a solvent for the preparation of the ceramic slurries. Diammonium hydrogen citrate (DAC, d.d.a. Sigma-Aldrich, St. Louis, MO, USA) and citric acid (CA, d.d.a. POCH Gliwice/Avantor Performance Materials Poland) were used as liquefiers. A 10% polyvinyl alcohol (PVA, St. Louis, MO, USA, Sigma-Aldrich) water-based solution was also used.

First, the dispersant, a composition of DAC and CA, was dissolved in an appropriate amount of distilled water. The PVA solution was then added. The amount of binder and dispersant added was selected experimentally for the individual slurries. In the next step, ceramic ZrO_2_ and Al_2_O_3_ powders were added. Four slurries with different ZrO_2_ volume contents (0%, 2.5%, 25%, 100%) were prepared during the study. Each prepared mass was subjected to mixing at 300 rpm for 1 h in a centrifugal Retsch (Haan, Germany) PM400 planetary mill in alumina containers. The slurry prepared in this way was vented at 2200 rpm for 11 min in a THINKY ARE-250 machine from Thinky Corporation (Tokyo, Japan). The vented mass was then poured into gypsum molds to form 16 mm × 8 mm cylinders in the raw state. The samples were then dried in a laboratory oven at 30 °C for 24 h. In the last step, the fabricated green bodies were sintered in a Nabertherm GmbH model HT 08/18 furnace equipped with superkanthal heaters in an air atmosphere according to the following program: temperature rise 2 °C/min to 1400 °C, holding for 2 h, cooling at 3 °C/min. Both powders have high sintering activity and can be sintered in relatively low temperature of 1400 °C. Thus, such temperature was selected for the sintering process.

Within the framework of the present work, four series of samples were made: Series I—0 vol.% of ZrO_2_, Series II—2.5 vol.% of ZrO_2_, Series III—25 vol.% of ZrO_2_, and Series IV—100 vol.% of ZrO_2_. The compositions of each series are shown in Table 1.

Thermogravimetric analysis was carried out to investigate the phenomena occurring in the fabricated ceramic fittings as a function of temperature at the assumed temperature process. The scope of the study included measuring mass loss and thermal effects as a function of temperature using Netzsch’s TG 449 F1 Jupiter thermogravimetric analyzer. Measurements were carried out in alundum crucibles. The green samples crushed in an agate mortar were the samples for analysis. The weight of the test portions was ~30 mg. Such small amounts of weighting guarantee excellent signals. The analysis was carried out in a flow of synthetic air (80/20 mixture of nitrogen and oxygen). The flow was constant and set at the value of 70 mL/min. Measurements were carried out in the temperature range from 24 to 1400 °C with a heating rate of 10 °C/min. The heating rate is much higher than the heating rate during the sintering process, which is because the signals in the Differential Thermal Analysis—DTA curve are more pronounced under these measurement conditions.

In contrast, the small sample size means that even at this rate of temperature increase, the temperature distribution in the sample is uniform. Automatic recording of sample weight loss and thermal effects was carried out during the measurement. Before measuring the samples, a calibration measurement was carried out on the empty crucibles, which were later used for testing. The calibration measurement was carried out under the same conditions and according to the same temperature program as the actual measurements to remove signals related to device effects. Thermogravimetric measurements as a function of temperature were then carried out.

Dilatometric tests were then carried out to determine the thermal expansion coefficients. The scope of the study included measuring changes in the linear dimensions of cylindrical specimens in the temperature range from 24 to 1000 °C with heating rate of 5 °C/min. A Netzsch dilatometer (model Dil 402C) (NETZSCH-Gerätebau GmbH, Selb, Germany) equipped with a quartz tube was used in this study.

The coefficient of thermal expansion was calculated from the results. The result obtained is the so-called physical expansion coefficient, i.e., the slope of the tangent to the curve of change in linear dimensions at one point over an infinitely small temperature interval and was calculated from Equation (1):

(1) $$\alpha \left(T\right)=\frac{dl}{dT}\cdot \frac{1}{l_{0}}$$

where *α* is the thermal expansion coefficient, *l* is the linear dimension of the sample at temperature *T*, and *l*_0_ is the linear dimension of the sample.

The technical coefficient of linear expansion of the samples in the temperature range was also determined as 100–500 °C using Formula (2):

(2) $$\alpha_{T_{1}}^{T_{2}}=\frac{\Delta l}{\Delta T}\cdot \frac{1}{l_{0}}=\frac{l_{T_{2}}-l_{T_{1}}}{T_{2}-T_{1}}\cdot \frac{1}{l_{0}}$$

where $\alpha_{T_{1}}^{T_{2}}$ is the technical expansion coefficient; $l_{T_{1}}$, $l_{T_{2}}$ are the linear dimensions of the sample at temperature *T*_1_ and *T*_2_, respectively; and *l*_0_ is the linear dimension of the sample.

Prior to dilatometric testing, the samples were sintered according to the sintering program described above in the section on fabricating samples. Prior to the measurements, the surfaces of the samples were ground using a grinder fitted with a diamond-coated disc to produce parallel surfaces.

Selected physical properties such as apparent density, relative density, and open porosity of the produced ceramic and ceramic composites were determined using the Archimedes method. In the experiment, the theoretical density of samples was determined on the basis of the rule of mixtures. Based on the helium pycnometer analyses of pure zirconia and alumina powders the theoretical density was calculated.

In this research, X-ray diffraction (XRD) tests were carried out using a Rigaku MiniFlex 2 (Rigaku Corporation, Tokyo, Japan) X-ray diffractogram with a copper lamp. Measurements were made over an angular range of 2θ = 20°–100° and with a step of 0.02° and a counting time of 1 s. Diffractometric data were processed using MDI JADE 7 software (MaterialsData, California, CA, USA). The ICDD PDF-4+ 2020 X-ray standard database was used to interpret the results. XRD studies were carried out for both the raw and sintered samples.

Microstructure analysis was performed based on fractographic observations. Fractographic analysis for fracture observation was performed using a JEOL scanning electron microscope model JSM-6610 (JEOL, Tokyo, Japan). The observations were carried out at an accelerating voltage of 15 kV. In order to obtain an image, it was necessary to cover the samples with a thin layer of carbon. For this purpose, a QUORUM Q150T ES sputtering machine was used (Headquarters, Laughton, East Sussex., UNITED KINGDOM).

Stereological analysis was performed to determine the effect of ZrO_2_ content on Al_2_O_3_ grain growth and grain shape parameters in the composites. The MicroMeter program [35,36] was used to perform the quantitative analysis. A description of the determination of shape parameters has been presented in previous works [37]. Furthermore, based on the image analysis of the microstructures of the composite samples (Series II and Series III), the specific areas of the Al_2_O_3_ phase boundaries were determined, which is characterized by the S_v_ parameter calculated by the MicroMeter program [35,36]. The unit of S_v_ is [1/µm]. This means that, by definition, S_v_ is the area of the phase boundaries in a unit volume [µm^2^/µm^3^] = [1/µm].

The environmental impacts associated with the production of Al_2_O_3_- and ZrO_2_-based ceramic samples were determined using the Life Cycle Assessment (LCA) method. The analysis was based on the guidelines of ISO 14044 [38], covering the sourcing and processing of raw materials—module A1—and the manufacture of product A3, as recommended for construction products in EN 15804 [39]. The calculation methodology adopted is based on mass allocation. All impacts associated with the generation of components such as ZrO_2_, Al_2_O_3_, DAC, CA, PVA, and distilled water were included in Module A1. The impacts associated with the generation and consumption of electricity to power the equipment used in the production of Al_2_O_3_/ZrO_2_ samples under laboratory conditions are included in Module A3. The electricity consumption was determined based on the operating time and manufacturers’ information on the capacity of the equipment. The waste volume was estimated as 1.5% of the initial mass loss after the venting process. The environmental impacts determined were expressed per sample, with the weight resulting from the formulation. Inventory data (LCI) and environmental indicators used for LCA calculations derived from the Ecoinvent v. 3.7, Environmental Product Declaration (EPD) and the emission factors contained in the Kobize 2020 report [40]. Table 2 shows the electricity consumption per Al_2_O_3_/ZrO_2_ specimen.

## 3. Results and Discussion

When forming ceramic bodies, an essential step is the sintering process of the samples, which should be selected individually for each type of material. This is made possible by thermogravimetric analysis, which examines the phenomena that accompany the heating of the samples to high temperatures and allows the selection of favorable conditions for the sintering process.

**Figure 1:**
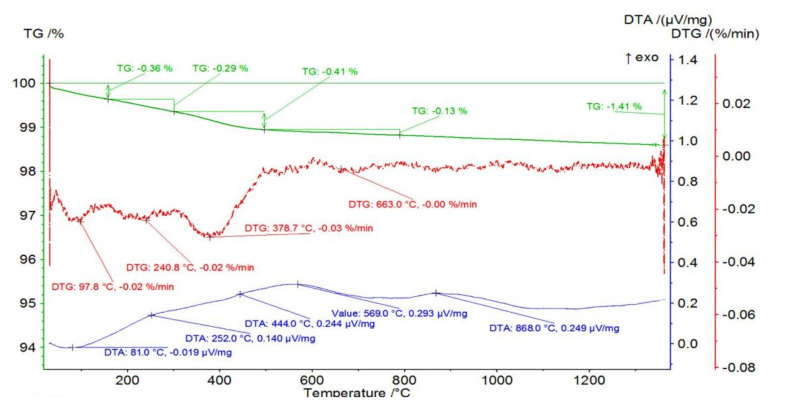
Mass change of the sample from Series I—0 vol.% of ZrO_2_ with thermogravimetric (TG) and differential thermal analysis (DTA) curves as a function of temperature.

Figure 1 shows the mass changes for an example sample from the Series I—0 vol.% of ZrO_2_ together with thermogravimetric (TG) curve and differential thermal analysis (DTA) curves as a function of temperature. From the curves obtained, it can be concluded that a total mass loss of 1.41% occurred in the sample containing 0 vol.% ZrO_2_ (100 vol.% Al_2_O_3_). In the first stage, a mass loss of 0.36% occurred in the temperature range RT-158 °C with an associated endothermic effect with a maximum at 97.8 °C. Endothermic effect and temperature range indicating the process of getting rid of physically bound water. This was followed by a mass loss of 0.29% in the temperature range of 158 to 301 °C. The maximum mass loss rate was recorded at 240.8 °C with an associated fuzzy exothermic effect with a maximum at 252.0 °C.

Similarly, a mass loss of 0.41% was recorded in the temperature range of 301 to 496 °C with a maximum mass loss at 378.7 °C and a fuzzy exothermic effect at 444.0 °C. A slight mass loss of 0.13% was observed in the temperature range of 496 to 788 °C with a maximum on DTG at 663.0 °C and an exothermic peak on DTA at 569.0 °C. At 868.0 °C, a broad, fuzzy peak is visible on the DTA, which is not accompanied by mass changes. The last two steps result from the oxidation of organic additives.

**Figure 2:**
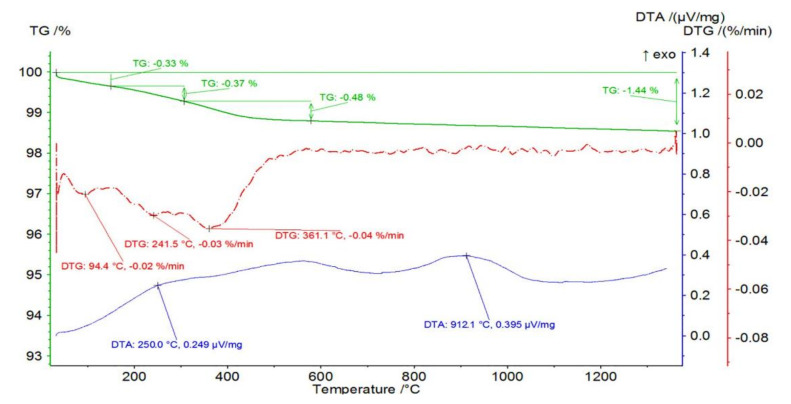
Mass change of the sample from Series II—2.5 vol.% of ZrO_2_ with TG and DTA curves as a function of temperature.

Figure 2 shows the mass change of an example sample from Series II—2.5 vol.% of ZrO_2_ together with the TG and DTA curves as a function of temperature. From these, it can be concluded that there was a total mass loss of 1.44% in the sample containing 2.5% ZrO_2_. In the first stage, a mass loss of 0.33% occurred in the RT-150 °C temperature range with an associated endothermic effect with a maximum at 94.4 °C with no apparent thermal effect. This was followed by a mass loss of 0.37% in the temperature range of 150 to 307 °C. The maximum mass loss rate was recorded at 241.5 °C with an associated fuzzy exothermic effect with a maximum at 250.0 °C. This step is probably due to the oxidation of organic additives. Similarly, a mass loss of 0.48% was recorded in the temperature range of 307 to 579 °C with a maximum mass loss at 361.1 °C and no apparent thermal effect. At 868.0 °C, a broad, fuzzy peak is visible on the DTA, which is not accompanied by mass changes.

**Figure 3:**
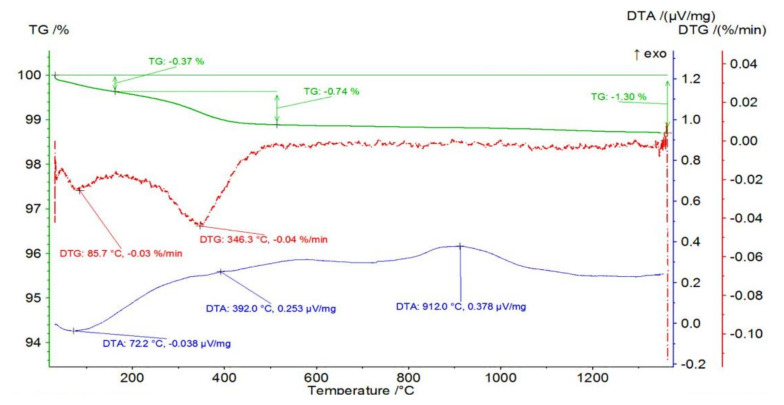
Mass change of the sample from Series III—25 vol.% of ZrO_2_ with TG and DTA curves as a function of temperature.

The mass changes occurring in an example sample from Series III containing 25 vol.% of ZrO_2_ were investigated in the next step. The obtained mass change of the sample from Series III—25 vol.% of ZrO_2_ with TG and DTA curves as a function of temperature is shown in Figure 3. The obtained results show that a total mass loss of 1.30% occurred in the sample containing 25 vol.% ZrO_2_. In the first stage, a mass loss of 0.37% occurred in the temperature range of RT-162 °C with an associated endothermic effect with a maximum at 72.2 °C. Endothermic effect and temperature range indicating a probable process of getting rid of physically bound water. This was followed by a mass loss of 0.74% in the temperature range of 162 to 514 °C. The maximum mass loss rate was recorded at 346.3 °C with an associated fuzzy exothermic effect with a maximum at 392.0 °C. The mass loss is probably due to oxidation of the organic additives. At 912.0 °C, a broad, fuzzy peak is visible on the DTA, which is not accompanied by mass changes.

**Figure 4:**
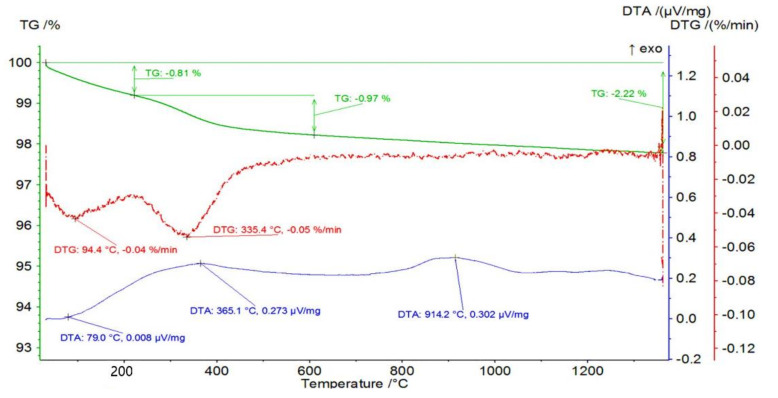
Mass change of the sample from Series IV—100 vol.% of ZrO_2_ with TG and DTA curves as a function of temperature.

Measurements of mass loss and thermal effects as a function of temperature were then determined for the Series IV—100 vol.% ZrO_2_ samples. The obtained curves of the mass change of an example sample from Series IV—100 vol.% of ZrO_2_ together with the TG and DTA curves as a function of temperature are shown in Figure 4. It was found that the sample containing 100% ZrO_2_ had a total mass loss of 2.22%. In the first stage, a mass loss of 0.81% occurred in the temperature range RT-222 °C with an associated endothermic effect with a maximum at 79.0 °C. Endothermic effect and temperature range indicating a probable process of getting rid of physically bound water. This was followed by a mass loss of 0.97% in the temperature range of 222 to 610 °C. The maximum mass loss rate was recorded at 335.4 °C with an associated fuzzy exothermic effect with a maximum at 365.1 °C. The mass loss is probably due to oxidation of the organic additives. At 914.2 °C, a broad, fuzzy peak is visible on the DTA, which is not accompanied by mass changes.

A summary of the thermogravimetric analysis is shown in Table 3. From an analysis of the results, it can be concluded that the first weight loss in all samples occurs up to 200 °C and is related to the removal of water. The evaporation temperature of the water is higher in this case as it is water physically bound to the components of the samples. Water molecules can be adsorbed on the surface of the powders and in the structure of the binder, which was PVA [41]. The amount of humidity in the samples is within the range of 0.36 to 1.44 wt%. This amount and the fact that the value is not stable indicates that the amount of water in the samples is dependent on the atmospheric conditions (ceramic powder adsorbs water from air).

During a further increase in temperature, the weight loss is related to the thermal decomposition of the organic elements present in the sample (DAC, CA, and PVA). Based on temperature reports, the individual stages of weight loss can be attributed to the thermal decomposition of organic additives. DAC decomposes in the temperature range of ca. 180–600 °C and occurs in two stages at ~180–250 °C and ~400–600 °C. Citric acid decomposes at a slightly lower temperature of 180–500 °C, and this process also occurs in two stages (180–250 °C and 250–500 °C) [42]. PVA decomposes in the temperature range of 200 to 450 °C. Analyzing the obtained results, it can be observed that as the ZrO_2_ content in the sample increases, the number of decomposition steps decreases, the organic substances are decomposed simultaneously, giving a single fuzzy signal on the DTA curve. This may indicate stronger interactions occurring between the powder surface and the organic matter adsorbed on it.

One of the fundamental physical properties of solids is thermal expansion; it plays a significant practical role. Changes in the volume of bodies due to temperature changes are crucial to the functioning of many devices, e.g., nozzle gaskets. Therefore, it is essential to determine the thermal expansion coefficients for the molded materials at the basic research stage. Thermal expansion is the change in volume of a solid due to a change in temperature (heating or cooling) while maintaining constant pressure. Note that the volumetric expansion of solids is most often simplified by reducing the problem by controlling changes in the body length.

**Figure 5:**
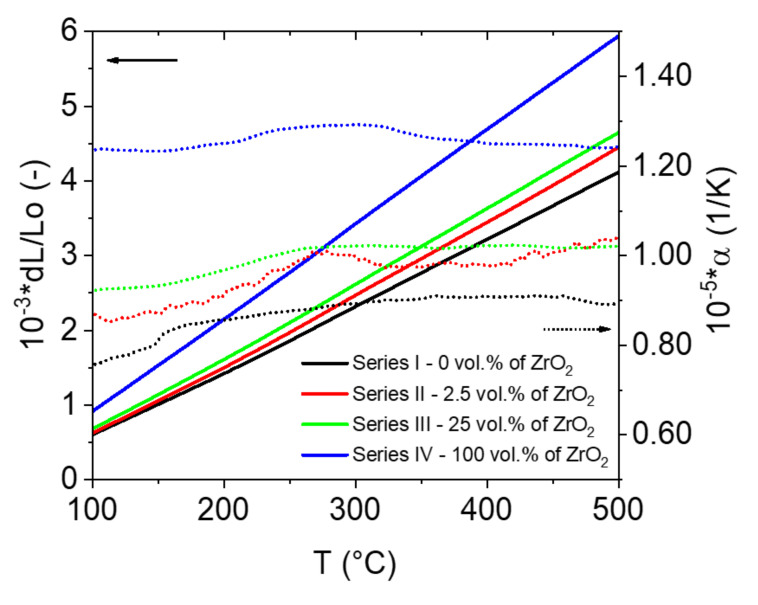
Chart of changes in the linear dimensions (solid lines) of the test specimens as a function of increasing and decreasing temperature with the physical coefficient of expansion (Equation (1); dotted lines).

Consequently, linear expansion is often assigned to solid substances as an indicator of thermal expansion. In the experiment conducted, this simplification was also used. Figure 5 shows the resulting changes in the linear dimensions of the samples as a function of increasing and decreasing temperature. The coefficient of thermal expansion was calculated from the results. The α factor for the alumina sample increases slightly with increasing temperature and is in the range of 8.24 to 9.22 × 10^−6^ 1/K. The presence of zirconium oxide in the alumina matrix, even in small quantities, causes a significant increase in the expansion coefficient. The determined α-factor for composites in the alumina-zirconium oxide system containing 2.5 vol.%, zirconium oxide is 8.05–10.5 × 10^−6^ 1/K. For composites containing 25 vol.%, zirconium oxide was found to expand in the range of 7.26 to 10.1 × 10^−6^ 1/K. In contrast, the expansion of a material made solely from zirconium oxide ranges from 10.9–12.6 × 10^−6^ 1/K.

**Figure 6:**
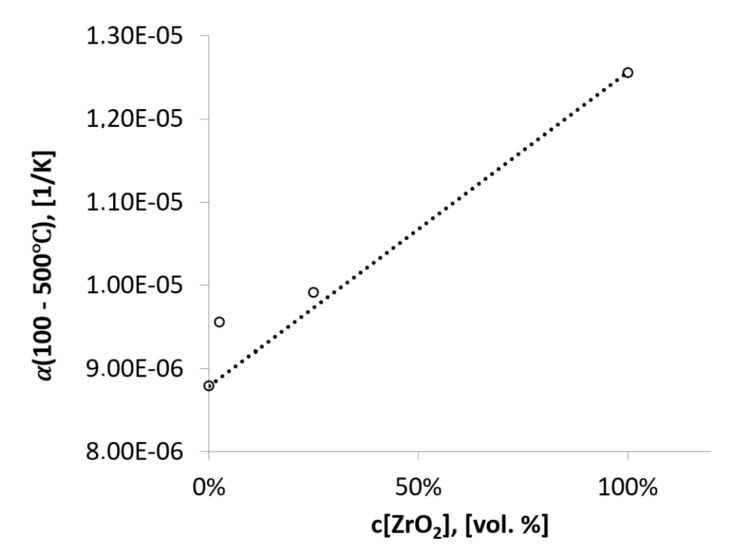
Technical coefficient of expansion in the temperature range 100–500 °C.

The values of the technical expansion coefficients in the temperature range of 100 to 500 °C are shown in Table 4. The values of the technical coefficient of expansion $\alpha_{T_{1}}^{T_{2}}$ are averaged values of the coefficient of expansion. The values of the coefficients $\alpha_{100{}^{\circ}C}^{500{}^{\circ}C}$ as a function of zirconium oxide content are shown in the chart in Figure 6. The chart shows the nonlinear nature of the dependence of the expansion coefficient on the concentration of zirconium oxide in the composite. Small amounts of zirconium oxide (2.5 vol.%) cause a jump in the coefficient $\alpha_{T_{1}}^{T_{2}}$ from 8.79 × 10^−6^ 1/K for the alumina sample to 9.56 × 10^−6^ 1/K for the composite containing 2.5 vol.% of zirconia. Such a phenomenon may indicate the presence of other phases in the composite (the composite is not a physical mixture). In contrast, composites containing 25 vol.% of zirconium oxide have a technical expansion coefficient value of 9.92 × 10^−6^ 1/K. This value is consistent with the linear nature of the coefficient changes (estimated from the law of additivity of properties).

Macroscopic observations in the sintered samples did not reveal visible defects, i.e., cracks or shape deformations. The densities of the specimen obtained are shown in Table 4. Based on the results, it can be concluded that the ceramic fittings from Series I—0 vol.% of ZrO_2_ and Series IV—100 vol.% of ZrO_2_ had a very high relative density above 99%. It was noted that the relative density values for the samples containing 100 vol.% of ZrO_2_ are slightly higher than the sample density values studied by Lada and her team [43]. In their work, they obtained samples (100 vol.% ZrO_2_) produced via slip casting with a density of 98%. The differences in values may be due to the type of ZrO_2_ powder used, the solid phase content of the slurry used, or the sintering process [43]. Based on the remaining density measurement results, the composites from the Al_2_O_3_/ZrO_2_ system (Series II and III) were found to have a lower relative density than samples containing only Al_2_O_3_ or ZrO_2_. From the results in Table 5, it is noted that for ZTA composites, changing the ZrO_2_ content in the sample does not significantly affect the change in relative density for Series II and Series III. The relative density for Series II and Series III composites were found to be 96.5% and 97%, respectively, which may indicate that mass transport in the composite was hindered due to the presence of another phase (ZrO_2_). Table 6 does not include the open porosity values for Series I and IV samples because the open porosity and water absorption are close to zero for samples with very high densities >99%. This is consistent with sintering theory because there should be no open pores at such high relative densities, so the absorbency should be zero.

**Figure 7:**
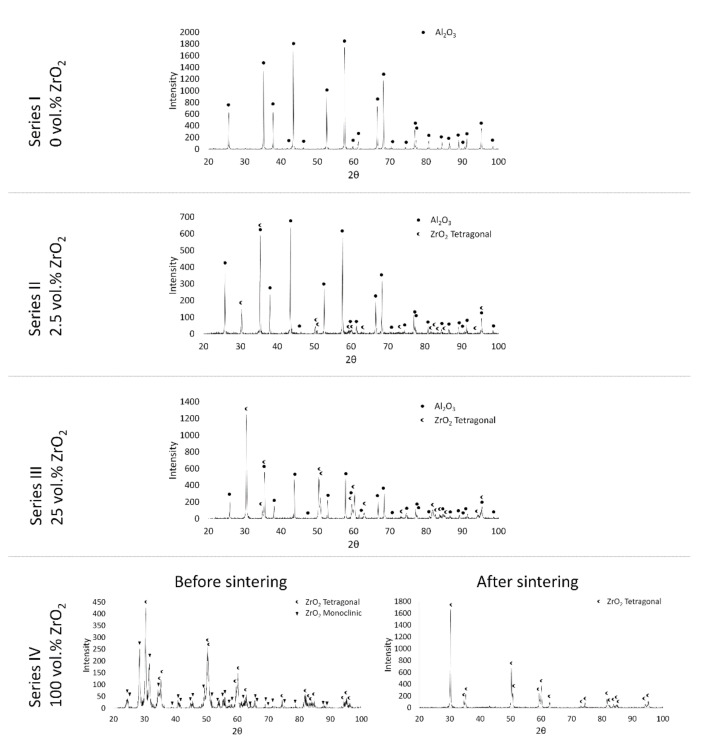
Diffractograms of samples fabricated via slip casting through and after the sintering process.

Figure 7 shows diffractograms of samples produced via slip casting through and after the sintering process with the main phases in the samples marked. From the phase analysis results obtained, it was observed that the diffractograms of Series I, II, and III samples after the sintering process do not differ significantly from the diffraction images for these materials before the sintering process. However, it is noticeable that there is an intense increase in the intensity of the individual peaks for the samples after the sintering process, which is due to the growth of grains and domains with a homogeneous crystalline structure. The XRD tests carried out showed that a sample containing 100 vol.% ZrO_2_ before the sintering process contained a monoclinic and a tetragonal phase. In the case of Series II and III composites, no monoclinic or regular phase was observed both before and after sintering, which may be due to the small amount of monoclinic variety in the whole sample. Sintering at 1400 °C resulted in a complete transformation of the monoclinic to tetragonal phase for series IV. Note that the presence of a tetragonal variety of ZrO_2_ increases the temperature stability of the material in operating conditions, e.g., as thermal barrier materials [44]. As early as the late 1970s and early 1980s, several researchers showed in their work that, for both solid materials and coatings, materials containing the tetragonal variety of ZrO_2_ exhibit greater mechanical strength, particularly against fracture, than those containing the monoclinic or regular variety [45,46,47].

**Figure 8:**
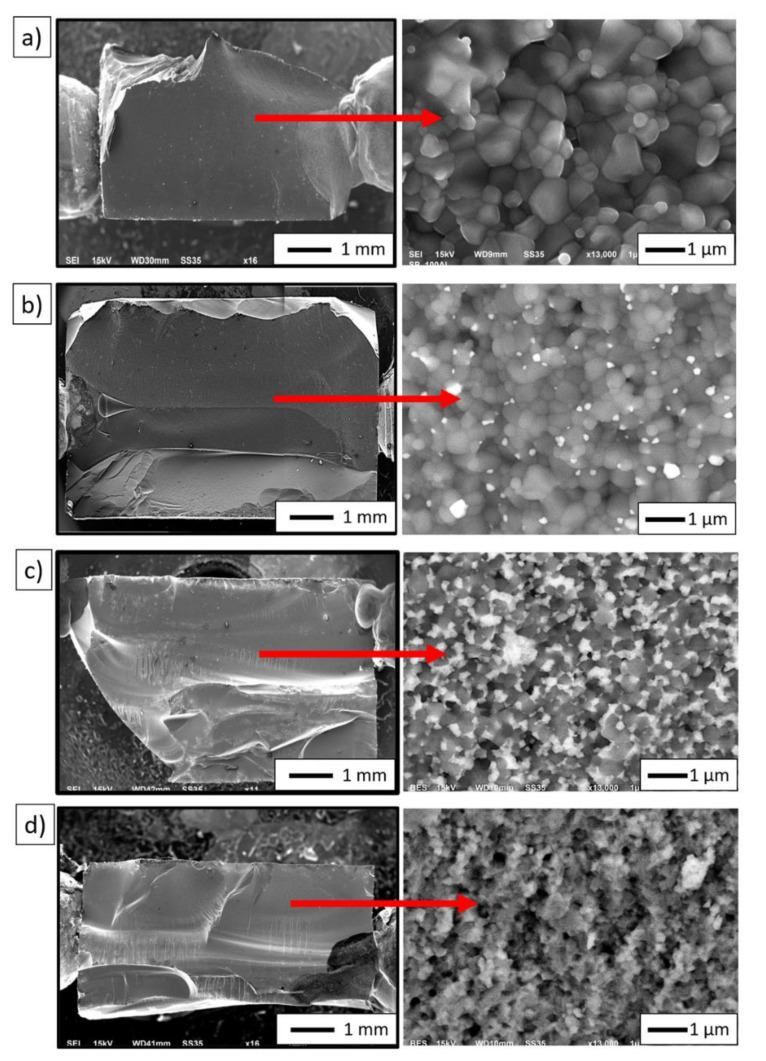
Fractographic observations of the samples produced by slip casting: (**a**) Series I—0 vol.% ZrO_2_, (**b**) Series II—2.5 vol.% ZrO_2_, (**c**) Series III—25 vol.% ZrO_2_, and (**d**) Series VI—100 vol.% ZrO_2_.

Figure 8 shows the results of a fractographic study carried out using a scanning electron microscope. In the images, the alumina grains are dark in color, while the zircon grains are lighter. Series I (100 vol.% Al_2_O_3_) is characterized by evenly spaced grains. In the Series IV sample (100 vol.% ZrO_2_), the grains are irregular in shape. A directional grain arrangement was also observed for the last series. From the results obtained, it was found that the main crack initiated and propagated on the surface of the specimens for all the materials analyzed. Cracking was observed to occur along grain boundaries in both Al_2_O_3_/ZrO_2_ composites and fittings containing 100 vol.% Al_2_O_3_ (Series I) and 100 vol.% ZrO_2_ (Series IV). This means that the cracks in the shapes studied are of an intergranular brittle nature. Furthermore, observations of the composite samples by scanning electron microscopy showed a uniform distribution of ZrO_2_ particles in the ceramic matrix throughout the test samples. Microstructural analysis of selected areas of the samples did not reveal the presence of areas characterized by excessive enrichment or depletion in ZrO_2_ particles regardless of the amount of zirconium oxide phase.

**Figure 9:**
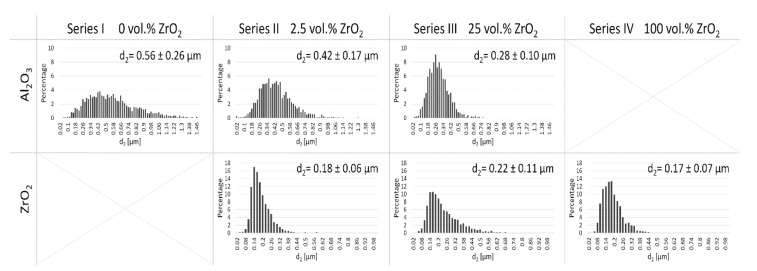
Histograms of grain distribution of Al_2_O_3_ and ZrO_2_ in individual series.

Image analysis was carried out in the next step based on the obtained microphotographs (Figure 8). This made it possible to determine the average grain size of Al_2_O_3_ and ZrO_2_ in the produced samples after the sintering process. The results obtained are shown in Figure 9. Based on the average grain size values obtained, it can be concluded that the addition of zirconium oxide effectively reduces the growth of alumina grains. In samples containing 100% vol. Al_2_O_3_ (Series I), the average grain size of Al_2_O_3_ was 0.56 µm. However, the addition of 2.5 vol.% ZrO_2_ (Series II) reduces Al_2_O_3_ grain growth by 25% (d_2_ = 0.42 µm) than the average Al_2_O_3_ grain size in samples containing only Al_2_O_3_. Interestingly, the addition of 25 vol.% ZrO_2_ reduces Al_2_O_3_ grain growth by 50% (d_2_ = 0.28 µm) compared to Al_2_O_3_ grains in samples containing 100 vol.% Al_2_O_3_. This is consistent with the density results obtained and the conclusion that the presence of ZrO_2_ grains in the matrix slightly hinders the densification of the Al_2_O_3_ matrix.

The results obtained are also in line with previous studies conducted with the same powder, using a different centrifugal slip casting method [34]. It was determined that for series II (2.5 vol.% ZrO_2_), the average grain size of ZrO_2_ was 0.18 µm, while for series III (25 vol.% ZrO_2_), the average grain size of ZrO_2_ was 0.22 µm. However, for samples containing only ZrO_2_, the average grain size of ZrO_2_ was 0.17 µm. Furthermore, the results obtained for ZrO_2_ grain size can also confirm the presence of a tetragonal phase in the sample. Literature data show that the tetragonal variety of ZrO_2_ is distinguished by a certain metastability depending on the grain characteristics of the material [46,48]. Patil and his research team showed that as early as the 1970s, the finer the grain, the greater the durability of the tetragonal phase in the material [48]. Adalberta and his research team came to similar conclusions in their study [46].

Table 6 shows the grain shape parameters of Al_2_O_3_ and ZrO_2_ in each series. Analysis of the results obtained showed that changing the ZrO_2_ content in individual batches does not affect the grain shape parameters of Al_2_O_3_ and ZrO_2_. The only deviation noted is the elongation of ZrO_2_ grains with increasing ZrO_2_ content in the samples.

The specific areas of the alumina phase boundaries in the Series II and Series III composites were then determined. Based on the results obtained, the specific surface areas of Al_2_O_3_ phase boundaries for series II—2.5 vol.% ZrO_2_ were 9.82 ± 0.22 [1/µm], while for series III—25 vol.% ZrO_2_ the S_v_ parameter was equal to 10.05 ± 0.56 [1/µm]. From the values obtained, it can be concluded that the Sv parameter decreases with increasing grain size of Al_2_O_3_ (Series II—d_2_ = 0.42 µm, Series III—d_2_ = 0.28 µm). This means that the surface area of the phase boundaries decreases with increasing ZrO_2_ content in the composite. This relationship is analogous to grain boundaries in metals. Namely, in metals, we can observe a relationship where the grain size is large, then the proportion of boundaries is small, and vice versa. In the composites studied, the proportion of interfacial boundaries is lower the larger the Al_2_O_3_ grains are.

Table 7 summarizes the Life Cycle Assessment (LCA) results of sintered Al_2_O_3_ and ZrO_2_ systems containing 0 vol.%, 2.5 vol.%, 25 vol.%, and 100 vol.% ZrO_2_, in terms of the acquisition and processing of raw materials—module A1, and their manufacture in the laboratory process—module A3. The results obtained indicate that increasing the ZrO_2_ content in the composite increases the value of the environmental footprint of the sample in the A1 phase, which has also been shown in studies [34,37]. This phenomenon is related to the lower availability of zirconium in the Earth’s crust and the higher energy and material intensity of the ZrO_2_ production process, compared to Al_2_O_3_ [49,50], which results in the fact that obtaining 1 kg of ZrO_2_ raw material, is accompanied by greenhouse gas emissions ranging from 3.4 kg eq. CO_2_, while for Al_2_O_3,_ it is 2.84 kg CO_2_ [51]. In manufactured composite materials, the greenhouse gas emissions in phase A1, per kg of sample, are in the order of 2.25 kg eq. CO_2_ and 2.43 kg eq. CO_2_ for a ZrO_2_ content of 2.5 vol.% and 25 vol.%, respectively. These impacts are comparable to the loads associated with the production of PVC or PP products [52,53]. As is well known, technologies are challenging to assess at an early stage of development. Making comparisons between different solutions, especially innovative and conventional solutions, is often complicated by differences in scale and resulting performance [54,55]. The analysis of the obtained LCA results of composites based on Al_2_O_3_ and ZrO_2_, fabricated via slip casting method, in the light of the authors’ previously published works on the preparation of analogous ceramic materials by the centrifugal slip casting method [34,37], proves that the choice of laboratory infrastructure is the crucial aspect influencing the environmental characteristics of the process [56]. The energy resource required to produce 1 kg of specimen by slip casting, using the infrastructure mentioned in the Materials and Methodology chapter, is 966 kWh, comparable to the electricity consumption of a hair dryer, used daily for 30 min, over a period of 2.5 years. On the other hand, the production of samples via the centrifugal slip casting method, which differs from the slip casting method by additionally using centrifugal casting, requires almost half the energy expenditure, amounting to 462 kWh per 1 kg of Al_2_O_3_ and ZrO_2_ specimens [34]. In this case, the size of the furnace chambers used for sintering the materials proved to be the determining factor for such a significant difference in the energy characteristics of the processes carried out under laboratory conditions.

## 4. Conclusions

Materials from the Al_2_O_3_ and ZrO_2_ system fabricated via slip casting were the subject of this study. The applied molding method made it possible to produce Al_2_O_3_ (Series I) and ZrO_2_ (Series IV) samples characterized by a relative density of >99%. The addition of ZrO_2_ to the Al_2_O_3_ matrix resulted in a lower density. The Al_2_O_3_/ZrO_2_ composites were found to have a density of about 97%. The samples were crack or defect-free after sintering. Phase composition analysis showed that the samples containing ZrO_2_ after the sintering process were characterized only by a tetragonal phase of ZrO_2_. The presence of a monoclinic or regular ZrO_2_ phase was not noted in the sample. Microscopic observations revealed a uniform distribution of ZrO_2_ in the Al_2_O_3_ matrix for Series II and III. Image analysis showed that the addition of ZrO_2_ effectively reduced the growth of Al_2_O_3_ grains. Furthermore, it was found that changing the ZrO_2_ content did not affect the shape parameters of Al_2_O_3_ grains after the sintering process. The results revealed that the amount of interfacial boundaries in Al_2_O_3_/ZrO_2_ composites decreases with increasing ZrO_2_ content in the samples.

Based on dilatometric tests, it was observed that the composites show a higher coefficient of linear expansion than that resulting from the content of the individual phases. The reason could be the porosity present in the composites, as samples containing 100 vol.% Al_2_O_3_ and 100 vol.% ZrO_2_ showed close to theoretical density, while the density of composites was 96.5 and 97%, respectively, for composite bodies containing 2.5 vol.% and 25 vol.% ZrO_2_. or the presence of other phases at the grain boundary. None of these factors can be ruled out at this stage of the work, and further research is required.

The results of the Life Cycle Assessment (LCA) have shown that the environmental burden (carbon footprint) resulting from the sourcing and processing of the raw materials required to produce sintered Al_2_O_3_ and ZrO_2_ is comparable to the burden associated with the production of plastic products such as PVC or PP. Note that the partial replacement of plastics with chemically inert ceramic material may contribute to reducing the increasingly perceived problem of the release of plastic microparticles and other harmful substances directly into soil and water. The study also shows that the development of new methods for the production of materials should take into account the assessment of environmental impact already at the conceptual stage, as the conscious choice of research infrastructure used in laboratory work can contribute to a reduction in the environmental footprint generated by the entire research environment.

## Figures and Tables

**Table 1:** Compositions of casting slips used to produce shapes via the centrifugal slip casting method.

	Total Solid Content	ZrO_2_	Al_2_O_3_	DAC	CA	PVA
	Vol.%	Vol.% with Respect to the Total Solid Volume		wt.% with Respect to the Content of ZrO_2_ and Al_2_O_3_		wt.% with Respect to the Total Solid Volume
Series I—0 vol.% of ZrO_2_	50	0	100	0.3	0.1	3
Series II—2.5 vol.% of ZrO_2_		2.5	97.5			
Series III—25 vol.% of ZrO_2_		25	75			
Series IV—100 vol.% of ZrO_2_		100	0			

**Table 2:** Life cycle inventory of Al_2_O_3_/ZrO_2_ composites formation via slip casting method.

Operation		Electricity Consumption per One Al_2_O_3_/ZrO_2_ Specimen
Homogenization in a planetary (ball) mill		140.0 Wh
Venting in the high-speed homogenizer		11.0 kW
Drying		11.2 kW
Sintering	Heating up to the set temp. (1400 °C)	875.0 kW
	Holding	150.0 kW
	Cooling	583.3 kW

**Table 3:** Summary of thermogravimetric analysis.

Sample Designation	Composition (Volume Fraction)		Temperature Range Including Weight Loss	Weight Loss Recorded	Temperature of Maximum Rate of Mass Loss	Temperature and Nature of the Heat Effect
	Al_2_O_3_	ZrO_2_				
Series I—0 vol.% of ZrO_2_	100%	0%	RT-158 °C	0.36%	97.8 °C	81.0 °C (endo)
			158–301 °C	0.29%	240.8 °C	252.0 °C (exo)
			301–496 °C	0.41%	378.7 °C	444.0 °C (exo)
			496–788 °C	0.13%	663.0 °C	569.0 °C (exo)
			-	-	-	868.0 °C (exo)
			RT-1400 °C	1.41%	-	-
Series II—2.5 vol.% of ZrO_2_	97.50%	2.5%	RT-150 °C	0.33%	94.4 °C	-
			150–307 °C	0.37%	241.5 °C	250.0 °C (exo)
			307–579 °C	0.48%	361.1 °C	-
			-	-	-	912.1 °C (exo)
			RT-1400 °C	1.44%	-	-
Series III—25 vol.% of ZrO_2_	75%	25%	RT-162 °C	0.37%	85.7 °C	72.2 °C (endo)
			162–514 °C	0.74%	346.3 °C	392.0 °C (exo)
			-	-	-	912.0 °C (exo)
			RT-1400 °C	1.30%	-	-
Series IV—100 vol.% of ZrO_2_	0%	100%	RT-222 °C	0.81%	94.4 °C	79.0 °C (endo)
			222–610 °C	0.97%	335.4 °C	365.1 °C (exo)
			-	-	-	914.2 °C (exo)
			RT-1400 °C	2.22%	-	-

**Table 4:** Technical coefficient of expansion.

Sample Designation	Composition (Volume Fraction)		Technical Coefficient of Expansion [1/K]
	Al_2_O_3_	ZrO_2_	in the Temp. Range 100–500 °C
Series I—0 vol.% of ZrO_2_	100%	0%	8.79 × 10^−6^
Series II—2.5 vol.% of ZrO_2_	97.50%	2.50%	9.56 × 10^−6^
Series III—25 vol.% of ZrO_2_	75%	25%	9.92 × 10^−6^
Series IV—100 vol.% of ZrO_2_	0%	100%	1.26 × 10^−5^

**Table 5:** Summary of density measurement results for shapes sintered at 1400 °C.

Sample Designation	Theoretical Density	Apparent Density	Relative Density	Open Porosity
	[g/cm^3^]	[g/cm^3^]	[%]	[%]
Series I—0 vol.% of ZrO_2_	3.98	3.95 ± 0.01	99.25	-
Series II—2.5 vol.% of ZrO_2_	4.03	3.89 ± 0.01	96.53	0.53
Series III—25 vol.% of ZrO_2_	4.46	4.33 ± 0.01	97.08	0.11
Series IV—100 vol.% of ZrO_2_	5.89	5.87 ± 0.01	99.66	-

**Table 6:** Shape parameters for Al_2_O_3_ and ZrO_2_ in individual series.

		Series I—0 Vol.% ZrO_2_	Series II—2.5 Vol.% ZrO_2_	Series III—25 Vol.% ZrO_2_	Series IV—100 Vol.% ZrO_2_
Al_2_O_3_	Curvature of grain boundary	1.22 ± 0.01	1.25 ± 0.01	1.24 ± 0.02	-
	Elongation	1.35 ± 0.02	1.36 ± 0.01	1.38 ± 0.02	-
	Convexity	1.07 ± 0.01	1.08 ± 0.01	1.07 ± 0.01	-
ZrO_2_	Curvature of grain boundary	-	1.12 ± 0.01	1.21 ± 0.02	1.24 ± 0.01
	Elongation	-	1.22 ± 0.02	1.34 ± 0.02	1.37 ± 0.01
	Convexity	-	1.03 ± 0.01	1.07 ± 0.02	1.07 ± 0.01

**Table 7:** Life cycle assessment results of sintered Al_2_O_3_- and ZrO_2_-based samples systems containing 0 vol.%, 2.5 vol.%, 25 vol.%, and 100 vol.% ZrO_2_.

Indicator	Unit	0 Vol.% ZrO_2_ (3.40 g)		2.5 Vol.% ZrO_2_ (3.43 g)		25 Vol.% ZrO_2_ (3.72 g)		100 Vol.% ZrO_2_ (4.68 g)	
		A1	A3	A1	A3	A1	A3	A1	A3
Global warming potential	kg CO_2_ eq.	7.59 × 10^−3^	1.40 × 10^0^	7.73 × 10^−3^	1.40 × 10^0^	9.03 × 10^−3^	1.40 × 10^0^	1.33 × 10^−2^	1.40 × 10^0^
Depletion potential of the stratospheric ozone layer	kg CFC 11 eq.	3.92 × 10^−10^	0.00 × 10^0^	5.00 × 10^−10^	0.00 × 10^0^	1.48 × 10^−9^	0.00 × 10^0^	4.73 × 10^−9^	0.00 × 10^0^
Acidification potential of soil and water	kg SO_2_ eq.	5.54 × 10^−5^	2.06 × 10^−3^	5.57 × 10^−5^	2.06 × 10^−3^	5.85 × 10^−5^	2.06 × 10^−3^	6.77 × 10^−5^	2.06 × 10^−3^
Formation potential of tropospheric ozone	kg Ethene eq.	3.13 × 10^−6^	0.00 × 10^0^	3.14 × 10^−6^	0.00 × 10^0^	3.22 × 10^−6^	0.00 × 10^0^	3.47 × 10^−6^	0.00 × 10^0^
Eutrophication potential	kg (PO_4_)^3-^ eq.	1.22 × 10^−5^	1.50 × 10^−4^	1.26 × 10^−5^	1.50 × 10^−4^	1.59 × 10^−5^	1.50 × 10^−4^	2.70 × 10^−5^	1.50 × 10^−4^
Abiotic depletion potential for non-fossil resources	kg Sb eq.	3.60 × 10^−8^	5.20 × 10^−6^	4.85 × 10^−8^	5.20 × 10^−6^	1.61 × 10^−7^	5.20 × 10^−6^	5.38 × 10^−7^	5.20 × 10^−6^
Abiotic depletion potential for fossil resources	MJ	8.81 × 10^−2^	1.40 × 10^1^	9.10 × 10^−2^	1.40 × 10^1^	1.17 × 10^−1^	1.40 × 10^1^	2.05 × 10^−1^	1.40 × 10^1^
Total use of renewable primary energy resources	MJ	2.64 × 10^−3^	1.54 × 10^0^	3.52 × 10^−3^	1.54 × 10^0^	1.15 × 10^−2^	1.54 × 10^0^	3.81 × 10^−2^	1.54 × 10^0^
Total use of non-renewable primary energy resources	MJ	7.20 × 10^−2^	1.47 × 10^1^	7.42 × 10^−2^	1.47 × 10^1^	9.39 × 10^−2^	1.47 × 10^1^	1.59 × 10^−1^	1.47 × 10^1^
Use of secondary material	kg	0.00 × 10^0^	0.00 × 10^0^	0.00 × 10^0^	0.00 × 10^0^	0.00 × 10^0^	0.00 × 10^0^	0.00 × 10^0^	0.00 × 10^0^
Use of renewable secondary fuels	MJ	0.00 × 10^0^	0.00 × 10^0^	0.00 × 10^0^	0.00 × 10^0^	0.00 × 10^0^	0.00 × 10^0^	0.00 × 10^0^	0.00 × 10^0^
Use of non-renewable secondary fuels	MJ	0.00 × 10^0^	0.00 × 10^0^	0.00 × 10^0^	0.00 × 10^0^	0.00 × 10^0^	0.00 × 10^0^	0.00 × 10^0^	0.00 × 10^0^
Net use of fresh water	m^3^	7.63 × 10^−4^	1.00 × 10^−2^	9.86 × 10^−4^	1.00 × 10^−2^	3.00 × 10^−3^	1.00 × 10^−2^	9.70 × 10^−3^	1.00 × 10^−2^
Hazardous waste disposed	kg	4.35 × 10^−8^	0.00 × 10^0^	4.82 × 10^−8^	0.00 × 10^0^	9.00 × 10^−8^	0.00 × 10^0^	2.29 × 10^−7^	0.00 × 10^0^
Non-hazardous waste disposed	kg	3.53 × 10^−3^	5.09 × 10^−2^	3.50 × 10^−3^	5.09 × 10^−2^	3.18 × 10^−3^	5.09 × 10^−2^	2.11 × 10^−3^	5.09 × 10^−2^
Radioactive waste disposed	kg	1.37 × 10^−7^	0.00 × 10^0^	1.48 × 10^−7^	0.00 × 10^0^	2.42 × 10^−7^	0.00 × 10^0^	5.58 × 10^−7^	0.00 × 10^0^
Components for re-use	kg	0.00 × 10^0^	0.00 × 10^0^	0.00 × 10^0^	0.00 × 10^0^	0.00 × 10^0^	0.00 × 10^0^	0.00 × 10^0^	0.00 × 10^0^
Materials for recycling	kg	0.00 × 10^0^	0.00 × 10^0^	0.00 × 10^0^	0.00 × 10^0^	0.00 × 10^0^	0.00 × 10^0^	0.00 × 10^0^	0.00 × 10^0^
Materials for energy recover	kg	0.00 × 10^0^	0.00 × 10^0^	0.00 × 10^0^	0.00 × 10^0^	0.00 × 10^0^	0.00 × 10^0^	0.00 × 10^0^	0.00 × 10^0^

## Data Availability

Data sharing not applicable.
